# ODM2CDA and CDA2ODM: Tools to convert documentation forms between EDC and EHR systems

**DOI:** 10.1186/s12911-015-0163-5

**Published:** 2015-05-26

**Authors:** Martin Dugas

**Affiliations:** Institute of Medical Informatics, University of Münster, Albert-Schweitzer-Campus 1, A11, D-48149 Münster, Germany

**Keywords:** EHR, EDC, CDA, ODM, Documentation form, Data model

## Abstract

**Background:**

Clinical trials apply standards approved by regulatory agencies for Electronic Data Capture (EDC). Operational Data Model (ODM) from Clinical Data Interchange Standards Consortium (CDISC) is commonly used. Electronic Health Record (EHR) systems for patient care predominantly apply HL7 standards, specifically Clinical Document Architecture (CDA). In recent years more and more patient data is processed in electronic form.

**Results:**

An open source reference implementation was designed and implemented to convert forms between ODM and CDA format. There are limitations of this conversion method due to different scope and design of ODM and CDA. Specifically, CDA has a multi-level hierarchical structure and CDA nodes can contain both XML values and XML attributes.

**Conclusions:**

Automated transformation of ODM files to CDA and vice versa is technically feasible in principle.

## Background

Inefficient and redundant documentation processes are a key problem in medical research. The documentation burden in clinical studies is huge and increasing: According to Getz [1]–based on an analysis of approximately 10.000 study protocols–the average amount of case report forms (CRFs) per patient in a clinical trial increased from 55 (1999-2002) to 180 pages (2003-2006). This surge is primarily caused by regulatory requirements, in particular regarding adverse drug events. High documentation workload is certainly a major cost factor for clinical research. It restricts recruitment of patients into clinical trials, simply because the number of available physicians is limited.

From an informatics point of view, routine documentation is performed in Electronic Health Record (EHR) systems. These are currently separated from Electronic Data Capture (EDC) systems for research purposes. From a process perspective, this leads to redundant data entry: Data from the same patient, regarding identical medical problems, are managed in separate systems. It is well known that uncontrolled redundancy is inefficient and causes data inconsistency. Integrating the Healthcare Enterprise (IHE) methods like IHE Retrieve Form for Data Capture (RFD) try to improve data integration, but are limited due to isolated systems. From a regulatory point of view, EHR systems are currently not validated for clinical trials–in contrast to EDC systems.

From a medical point of view, the overall purpose of high-volume documentation in EDC systems is very similar to EHR systems: The physician provides detailed reports about diagnostic and therapeutic actions. In the clinical context, this is needed to communicate within the clinical team and to provide evidence for treatment according to state-of-the-art. In a study context, detailed documentation is demanded to identify adverse events as early as possible.

It is necessary to analyze, compare and potentially harmonize data structures in EHR and EDC systems to address this problem of redundant documentation in EHR and EDC systems.

Quite different standards evolved because EHR and EDC systems are separated. At present, clinical document architecture (CDA) from HL7 [2] is the most established industry standard in EHR systems. It is approved by the American National Standards Institute (ANSI) and supported by major EHR vendors worldwide. Regarding EDC systems, standards reflect requirements from regulatory agencies, in particular Food and Drug Administration (FDA [3]) and European Medicines Agency (EMA [4]). Currently standards from the Clinical Data Interchange Consortium (CDISC [5]) are most accepted industry standards for EDC systems. More specifically, CDISC’s Operational Data Model (ODM) is available to represent CRFs. ODM was introduced in 1999, the current version is 1.3.2. It is a platform independent format for exchange of clinical trial metadata and data. ODM is adopted by many EDC vendors (for example Medidata Rave®).

From a user’s point of view, data collection with electronic forms is a laborious task, both in EHR and EDC systems. These contain 1.800+ data elements on average (180 pages per trial, 10+ items per page). In the past, automated conversion between different technical representations of these data elements was not available. The scope of conversion is on the metadata level, i.e., conversion of “empty” forms (list of data elements, no patient-level data).

The objectives of this work are:

1. To provide a transformation tool for data structures of CRFs, represented in CDISC ODM, into HL7 CDA format, thereby enabling to integrate CRF data structures into EHRs,
2. to provide a converter for EHR forms into EDC format, specifically HL7 CDA into CDISC ODM format and
3. to describe the limitations of this conversion process.

## Implementation

Firstly, the meaning of “documentation form” will be explained, then a short explanation of the standards CDISC ODM and HL7 CDA will be provided. Secondly, the mapping process between ODM and CDA will be introduced with more details. Thirdly, an evaluation approach to test the transformation will be presented as well as some technical aspects of the reference implementation.

### Documentation form

A documentation form is considered a list of data items. Each data item is characterized by a name, for example “patient weight”, and a data type, such as “float number”. This simple representation of forms is commonly used in statistical programs like IBM SPSS [6]. Additional properties of forms are not taken into account, such as layout information, business logic, font type or location coordinates of data items.

### CDISC ODM

**Fig. 1:**
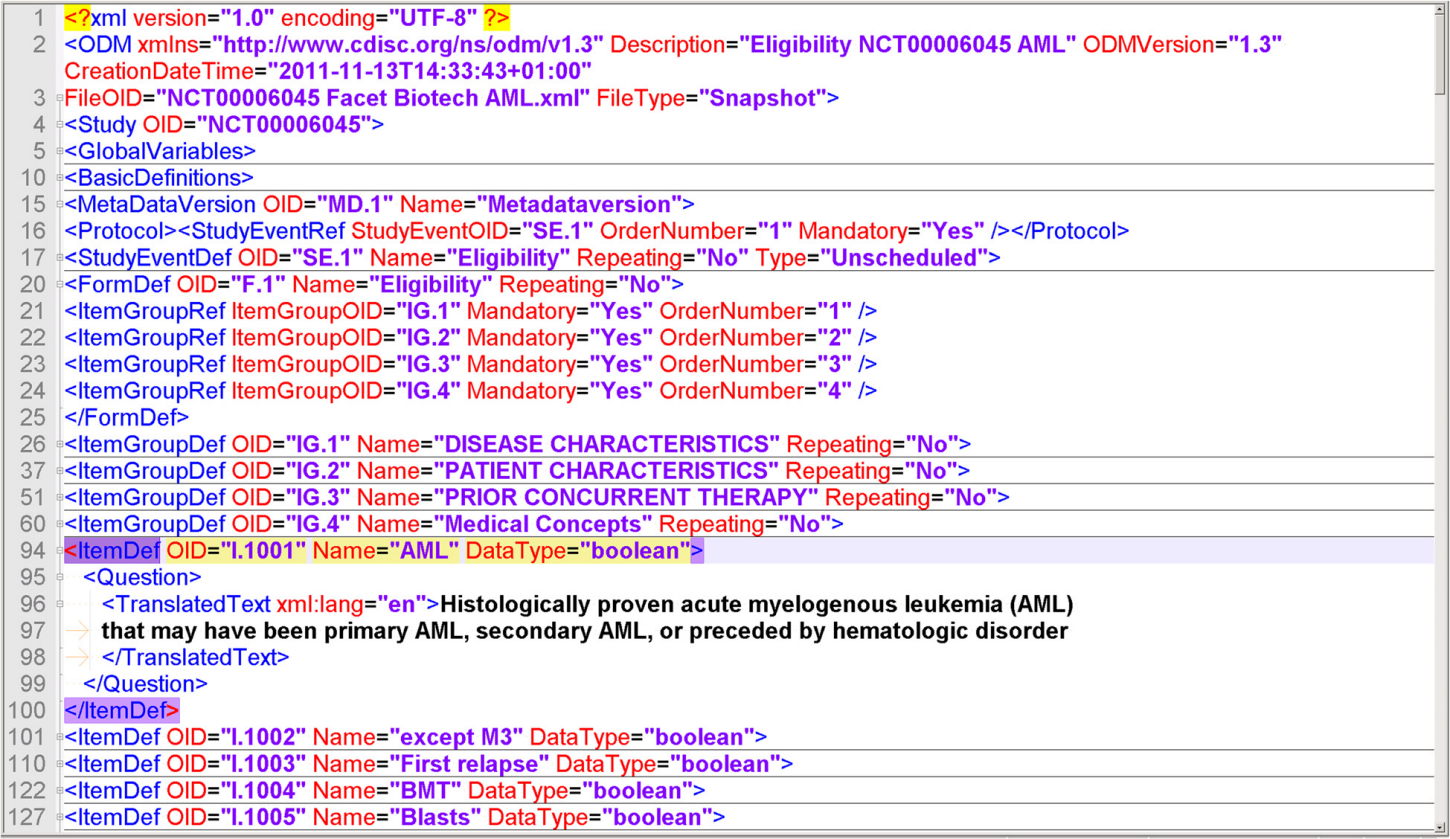
Example of ODM file. A CRF with 4 itemgroups (IG.1 - IG.4) is presented. Details for item I.1001 are provided such as data type and elaborated text

CDISC ODM [7] is an XML-based transport format for both metadata and data in clinical trials. It is applied in many EDC systems, which are accepted for clinical trials by FDA and EMA. Specifically, ODM enables to represent CRFs. A form is structured into item groups. Each item group consists of items. For each item, a data type and an optional code list can be specified. Fig. 1 presents an example of a form in ODM format.

### HL7 CDA

HL7 CDA [2] is an XML-based industry standard for exchange of clinical documents, for example discharge summaries or assessment forms. Basically, CDA can be used for any type of clinical content. Each CDA document consists of a header (descriptive metadata and data, for example title of document, creation time) as well as a body (structural metadata and data, for instance diagnosis codes). CDA includes a specification of document semantics and is based on the HL7 reference information model (RIM) [8]. CDA Release two has been adopted as ISO standard ISO/HL7 27932:2009 [9]. Dedicated CDA templates are defined by the HL7 organisation, i.e., not all CDA structures can be considered CDA templates.

**Fig 2.**
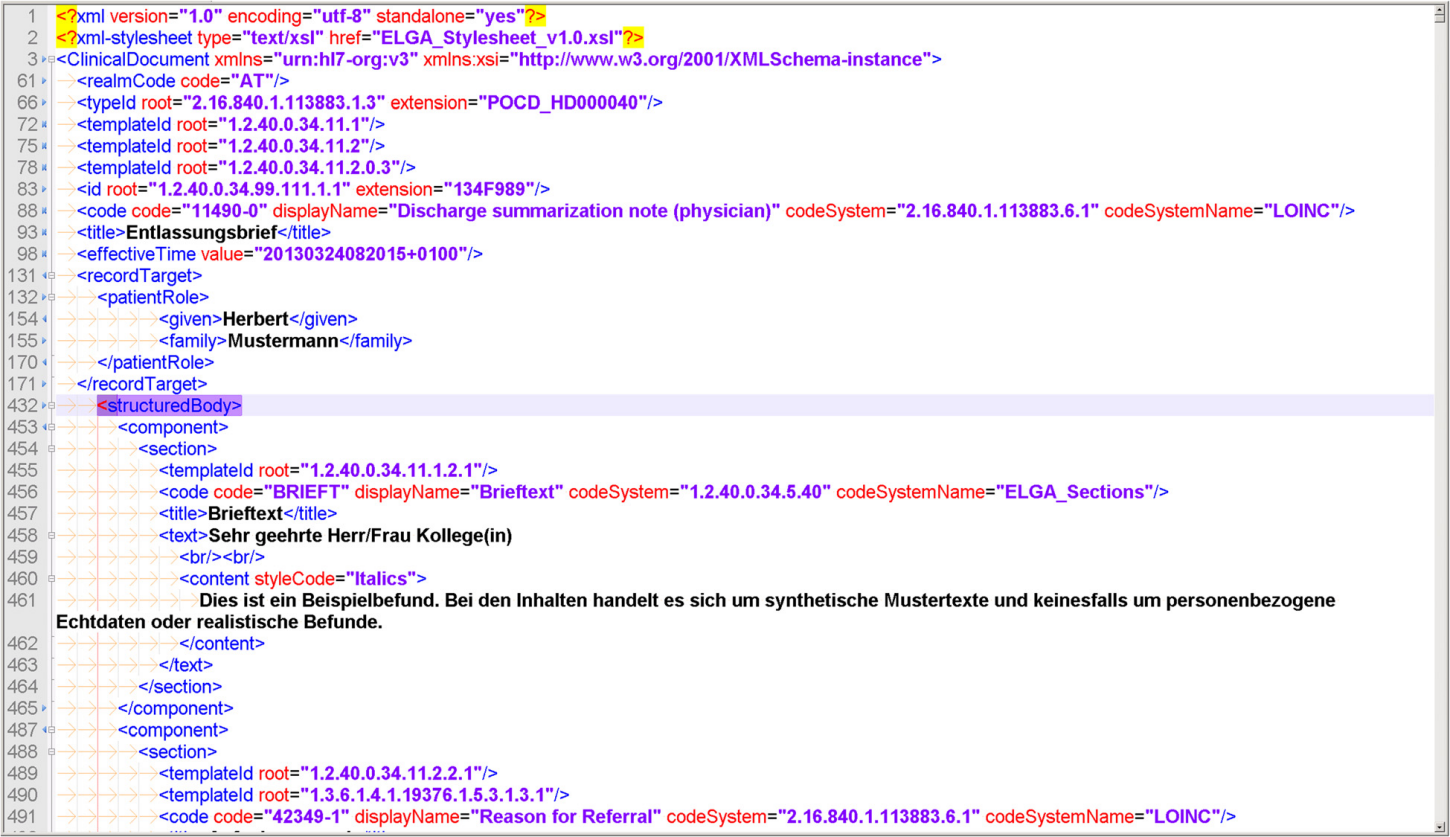
Example CDA file. A small extract from a discharge summary file is presented. The header contains a document code (“11490-0”), time stamp (“20130324082015 + 0100”) and patient name (“Herbert Mustermann”). The structured body consists of several components, starting with a section of discharge letter text (“Brieftext”)

Figure 2 presents an example of a CDA document from the Austrian electronic health record project [10].

### Mapping process ODM to CDA format

**Fig. 3:**
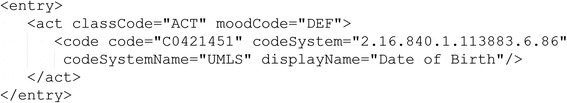
Example for an entry element of a CDA file. It corresponds to an ODM item named “Date of Birth”. C0421451 is the UMLS code for Date of Birth

XML elements in the CDA standard were reviewed to identify suitable data structures for CRF items. A CDA document consists of several sections, including a generic assessment section. Because a CRF can be considered a patient assessment, it was decided to map each CRF in ODM format into an assessment section of a CDA document. Each data item from a CRF was mapped to an entry element within this CDA assessment section (see Fig. 3). CDA entry elements can contain semantic annotations like Unified Medical Language System (UMLS) codes [11].

### Mapping process CDA to ODM format

ODM is designed to store patient data in item nodes, which are organised by itemgroups. In contrast, CDA uses different types of nodes and attributes to store patient data. The CDA standard was reviewed to identify XML nodes and XML attributes representing patient data.

The mapping process CDA to ODM is more complicated, because CDA nodes are nested: For example, the node < patient > within the CDA header contains a subnode < name>, which contains subnodes < given > and < family>. The name of the leaf node < given > is not necessarily unique. For this reason it was decided to generate unique ODM item names by concatenating node names from leaf nodes and parent nodes. ODM item names “patient.name.given” and “patient.name.family” would be generated in this example.

CDA uses both XML node values and XML attributes to represent patient data. For example, the home town of the patient can be stored as < city > Münster</city > and the E-mail address can be stored as < telecom value = “mailto:mustermann@mail.de”>. There can be several XML attributes per node (e.g., patient telephone number as another attribute), therefore the number of required ODM items cannot be directly determined from the number of CDA nodes. A suffix was added to the ODM item name for each attribute to generate unique names. For example: “telecom.attributes.value” as ODM item name for the attribute “value” of the CDA node < telecom >.

### Evaluation approach

To test the transformation of ODM to CDA, ten ODM files were extracted from the portal of medical data models [12]. These ODM files were converted into CDA format and the result was reviewed regarding syntactical correctness and limitations of the transformation.

To assess the conversion of CDA to ODM, ten public CDA files from the Austrian electronic health record project [10] were converted into ODM format. The transformation result was checked for schema conformance. This is necessary, but not sufficient for ODM validity (additional constraints need to be considered). Therefore transformed files were manually reviewed.

### Reference implementation

An open source reference implementation for conversion of documentation forms between ODM and CDA format was developed. ODM2CDA and CDA2ODM are implemented in R [13] and available with program documentation at http://cran.r-project.org within package ODMconverter.

In addition, ODM2CDA was implemented as web service and is available to the scientific community as download option within the portal of medical data models [14].

## Results

### Transformation of ODM to CDA

Table 1 provides details about ten ODM files which were converted into CDA format. Forms from different documentation settings such as routine documentation (e.g., HIS review of systems, Follow Up), clinical trials (e.g., Adverse Event AML-AZA) or clinical registries (e.g., Finnish cancer registry) were assessed. These forms are available at https://medical-data-models.org/forms/ {form number}.

**Table 1:** Basic characteristics of ODM files used for transformation testing

Form number	Form name	Number of data items
4589	Knochenmark AML-AZA	11
4590	Adverse Event AML-AZA	14
4701	Finnish Cancer Registry	49
4810	CDASH Vital Signs	14
5043	Eligibility AML-AZA NCT00915252	24
5165	Follow Up	16
5167	NCI Standard Adverse Event CTCAE v3 Template	28
5215	HIS review of systems	107
5216	EHR4CR data inventory	75
6413	AML-AZA Ersterhebung	94

Using the reference implementation of ODM2CDA, all ten forms were converted into CDA format. Conformance with CDA schema was tested successfully according to a public CDA XML schema definition [15]. In addition, these generated CDA files were manually reviewed to assess validity. Of note, these files are valid CDA structures, but no CDA templates, because these are defined by the HL7 organisation.

The transformation tool ODM2CDA was integrated as a web service into the portal of medical data models [14], therefore all available forms (>9.000) can be exported in CDA format.

It can be concluded that automated conversion from ODM to CDA format is technically feasible in principle. However, all ODM items are represented as CDA assessment items and other CDA sections are not taken into account for this transformation. In addition, ODM itemgroup information is not represented in CDA format.

### Transformation of CDA to ODM

Ten public CDA files (see Table 2) from the Austrian electronic health record project [10] were converted into ODM format. The transformation result was tested for syntactical correctness according to CDISC ODM (version 1.3.2) [7]. These converted CDA files were reviewed manually. Of note, these converted CDA files contain a large number of items (between 359 and 3013).

**Table 2:** Transformation results of ten CDA files from the Austrian electronic health record project

CDA file	Number of ODM data items
Findings imaging diagnostics (full support)	528
nurse discharge letter (basic)	359
nurse discharge letter (enhanced)	597
nurse discharge letter (full support)	755
physician discharge letter (basic)	359
physician discharge letter (enhanced)	668
physician discharge letter (full support)	1087
physician discharge letter (full support minimal)	559
physician discharge letter (structured)	359
lab findings (full support)	3013

In addition, the converted CDA file “physician discharge letter (basic)” was uploaded to the portal of medical data models [14] and is available at https://medical-data-models.org/forms/5217.

This transformation has several limitations: The hierarchical structure of CDA (see methods section “Mapping process CDA to ODM format”) was approximated by concatenated ODM item names, for example “patient.name.family”. CDA nodes can contain both XML values and XML attributes, which need to be represented by separate ODM items. In addition, re-transformation of this ODM file into CDA does not generate the original CDA file: ODM2CDA stores ODM items only within the CDA assessment section.

## Discussion

Currently, the documentation processes for routine patient care and clinical research are disconnected. High regulatory requirements result in costly documentation processes [16]. Different technical standards are used in medical IT systems: Predominantly CDISC standards for clinical research and HL7 standards for EHR systems, in particular CDA. CDISC ODM is highly adopted by EDC vendors. ODM is used for data and metadata export, but recently more and more for metadata import. Regulatory authorities like FDA support CDISC standards, which is an important driver for this trend.

From a medical point of view, an EHR system and an EDC system collect information for the same patient. From a data analysis perspective, many data points in both systems–such as body weight–should be very similar or the same, only stored in different representations. CDISC and HL7 standards serve different purposes, so there will be differences between ODM and CDA.

Regarding data exchange between EHR and EDC systems, it would be very useful to extract data points from one representation and transform it into another one. A prerequisite for such data exchange is a transformation of ODM data structures into CDA and vice versa. This enables clinicians and researchers to identify similarities and differences between EHR and EDC documents. Comparison between EHR and EDC data structures can be used at the trial design stage to optimize EHR and EDC documentation. In the trial execution phase it can be used to identify data elements for re-use.

Transformation of ODM to CDA was already described in the literature [17], however so far no implementation was available to the scientific community. In this work we present an open source transformation program between ODM and CDA data structures. It was tested for two sets of files: ten ODM files from different documentation settings and ten public CDA files.

It was demonstrated that an automated transformation between ODM and CDA is technically feasible in principle. However, this transformation is “lossy”, i.e., has several limitations due to specific properties of ODM and CDA: ODM is much more generic, because it assigns data items to item groups, and these item groups to forms. In contrast, CDA consists mainly of predefined sections of XML nodes related to diagnosis, allergies, medication, findings etc. Many CDA documents contain a lengthy header with very detailed descriptive metadata regarding administrative patient data (name, address), physician and hospital-related data. From a data analysis perspective, most of these CDA header elements are not useful for clinical research questions. In contrast, the body section of many CDA files–which contains the interesting clinical data–is very short. From a technical perspective, processing CDA files is more complicated than ODM files: CDA combines XML node values with XML attributes and has a variable hierarchical structure.

There are several limitations of the proposed transformation between ODM and CDA.

In general, CDA is generated from data instances and it is not clear what data elements are optional or repeatable (by default, the conversion tool assigns attributes Mandatory = “Yes” and Repeating = “No”). CDA also provides narrative parts, i.e., non-structured data. In contrast, ODM defines a full schema with optional, mandatory and repeatable data elements. ODM items are represented as CDA assessment sections in the current implementation of the conversion tool. The hierarchical structure of CDA is approximated by concatenated item names in ODM format. ODM does not provide information about item classes, therefore act classes are generated in CDA. Narrative text from CDA is ignored when it cannot be assigned to structured data elements. The transformation is designed for metadata: CDA files contain data for one patient while ODM files can contain data for large patient cohorts.

Many differences between EHR and EDC standards have been reported [18], and there is a long scientific debate about standardised medical data models.

Transformation and mapping between EHR and EDC standards is a first, but important step to enable comparison and discussion of data items. From a data analysis perspective, a list of data items is a prerequisite for statistical analysis.

This requirement is addressed very well by the ODM standard. CDA was designed to represent the current heterogeneity of clinical data structures. From a methodological point of view, the large diversity of clinical documentation indicates that there is room for improvement by standardisation: It is highly unlikely that all the diverse documentation approaches are optimal. Transparency of data models and transformation between different standards like ODM and CDA are first steps to trigger a discussion about best practice in clinical and research documentation.

The proposed transformation approach can take into account semantic codes for data items. However, most publicly available medical forms are not (yet?) semantically annotated. The high number of data elements per documentation unit (up to 3000) in this study indicate a need for automated metadata processing (for instance [19]), because manual mapping of many data elements is resource-intensive and error-prone.

## Conclusion

Automatic transformation of ODM files to CDA and vice versa is technically feasible in principle, but has limitations due to the different scope of ODM and CDA standards. An open source reference implementation is available.

## Availability and requirements

**Project name:** ODMconverter

**Project home page:**http://cran.r-project.org/web/packages/ODMconverter/index.html

**Operating System:** Platform independent

**Programming language:** R

**Other requirements:** R packages XML and xlsx

**License:** GPL

**Any restrictions to use be non-academics:** N/A
